# BACTERIAL RESISTANCE TO ANTIBIOTICS IN ACNE VULGARIS: AN *IN VITRO* STUDY

**DOI:** 10.4103/0019-5154.43213

**Published:** 2008

**Authors:** Parvin Hassanzadeh, M Bahmani, Davood Mehrabani

**Affiliations:** *From the Department of Biology, School of Sciences, Shiraz University, Shiraz, Iran*; 1 *From the Department of Dermatology and Pathology, Faghihi Hospital, Shiraz, Iran*; 2 *From the Department of Gastroenterohepathology Research Center, Nemazee Hospital, Shiraz University of Medical Sciences, Shiraz, Iran*

**Keywords:** *Acne vulgaris*, *antibiotic resistance*, *southern Iran*, *rifampin*

## Abstract

**Background:**

Acne vulgaris is one of the most common skin disorders in youth especially during the puberty.

**Objective:**

This *in vitro* study was performed to determine the antibiotic resistance and sensitivity in acne vulgaris.

**Materials and Methods:**

Samples were collected from normal skin and nodulocystic and pustular skin lesions of one hundred youngsters (64 girls, 36 boys) among college students in the age range of 18-24 years old. The specimens were cultured individually on blood agar and Muller-Hinton media. The cultures were then incubated under both aerobic and anaerobic conditions for 2 to 7 days. Bacteria were identified and their resistance to common antibiotics was evaluated according to the standard procedures.

**Results:**

In aerobic culture of pustular and nodulocystic skin lesions, *Staphylococcus aureus* was present in 41% of subjects, *Staphylococcus epidermidis* in 53% and *Micrococcus spp* in 45% of subjucts. In anaerobic bacterial culture of pustular and nodulocystic skin lesions, *Staphylococcus aureus* was present in 39%, *Propionibacterium acne in* 33% and *Staphylococcus epidermidis* in 21% of subjects. The results of present study revealed that clindamycin and erythromycin were the least effective antibiotics for *Propionibacterium acne* while tetracycline was the least effective for *Staphylococcus aureus in vitro*. A synergic effect of benzoyl peroxide, erythromycin or clindamycin was noticed. Rifampin was the most effective antibiotic *in vitro*.

**Conclusion:**

Our results showed that rifampin was the most sensitive antibiotic *in vitro* for acne vulgaris. To achieve a better treatment, a combination of rifampin with other antibiotics may be more efficient. We suggest *in vivo* studies for better evaluation and treatment of acne patients with rifampin.

## Introduction

Acne vulgaris is a chronic inflammatory disorder of pilosebaceous follicles that affects more than 85 percent of adolescents and young adults.^1^,^2^ Four major factors are involved in the pathogenesis including increased sebum production, hypercornification of the pilosebaceous duct, an abnormality of the microbial flora especially colonization of the duct with *Propionibacterium acnes*), and the production of inflammation.^3^ It seems that several factors influence acne including diet, menstruation, sweating, stress, ultra violet radiation and occupation.^4^ Positive association between intake of milk and acne was reported and this findings support earlier studies and suggests that the metabolic effects of milk are sufficient to elicit biological responses in consumers.^5^ It was also reported that a low-glycemic-load diet improves symptoms in acne vulgaris patients.^6^ Acne is not an infectious disease, but three major organisms were isolated from the surface of the skin and the pilosebaceous duct of patients with acne including *Propionibacterium acne*, *Staphylococcus epidermidis* and *Malasezia furfur*.^3^ Depending on the severity of the disease, the acne patients receive topical or systemic therapy, or a combination.^7^,^8^ Pathogenesis of microorganism originates from production of proinflammatory mediators (e.g. IL-1, TNFα) as well as many lipases. Increased number of *Propionibacterium acne* was reported in acne patients, but their number was not correlated with the clinical severity.^9^ Due to development of a resistance in microorganisms causing acne to common antibiotics and the differences in species and strains of the microorganisms in different regions, a research in the method of therapy seems indispensable.^10^,^11^ This study was undertaken to determine bacteria involved in acne vulgaris in Shiraz, southern Iran and to clarify the *in vitro* antibiotic sensitivity in acne vulgaris.

## Materials and Methods

Samples from the normal skin and pustular and nodulocystic skin lesions were provided in 100 youngsters (64 girls, 36 boys) among college students, in the age range of 18-24 years. The subjects were asked to refer to Department of Biology of School of Sciences, Shiraz University from October 2004 to March 2005. The samples were immediately cultured individually on blood agar and Muller-Hinton media. The cultures were then incubated at 37°C under both aerobic and anaerobic conditions for 2 to 7 days. The colonies species were determined morphologically by specific culture media such as mannitol, indole and sorbitol media and specific standard microbial tests such as oxidase, catalase, and coagulase tests.^12^ The sensitivity of bacteria to antibiotics was determined according to the method of Kirbauy.^12^

## Results

The microorganisms in the microflora of the normal skin and pustular and nodulocystic skin lesions were grown both aerobically and anaerobically as presented in Table 1. A significant higher percentage of *Staphylococcus aureus* was observed in normal skin of both girls and boys compared to *Propionibacterium acne* in pustular and nodulocystic skin lesions (*P* < 0.05). The different bacteria in pustular and nodulocystic skin lesions in both genders were grown aerobically and anaerobically. Aerobically, *Staphylococcus aureus*, *Staphylococcus epidermidis*, *Propionibacterium acne* and *Micrococcus spp* were detected in 41%, 53%, 0%, 45% of samples respectively while these figures anaerobically were 39%, 21%, 33% and 0% respectively. When the effects of different antibiotics on *Propionibacterium acne*, *Micrococcus spp, Staphylococcus epidermidis, and Staphylococcus aureus* were tested (Table 2), *Propionibacterium acne*, *Staphylococcus epidermidis* and *Staphylococcus aureus* were more sensitive to rifampin compared to other drugs (*P* = 0.004). As shown in Table 2, the combined inhibitory effect of clindamycin or erythromycin with benzoyl peroxide was less than rifampin alone. Table 2 shows the effects of different antibiotics on isolated bacteria from pustular and nodulocystic skin lesions. The diameter of inhibition zone (mm) by each antibiotic was studied. According to the manual instruction of Padtan Tab Co., inhibition zone less than 17 mm^®^ was considered as resistance to antibiotic.

**Table 1:** Analysis of bacteria in samples obtained from youngsters [*n* = 100]

Cultures	Samples	*S. aureus*	*S. epidermidis*	*P. acne*	*Micrococcus spp*
Aerobic	Pustular and nodulocystic skin lesions (*n*)	51	53	-	45
	Microflora of normal skin (*n*)	30	73	-	32
Anaerobic	Pustular and nodulocystic skin lesions (*n*)	39	21	33	-
	Microflora of normal skin (*n*)	33	48	11	-

*n*: number; Percentages were calculated based on one hundred persons

**Table 2:** The effects of different antibiotics on isolated bacteria of acne vulgaris

Name of antibiotic	Sensitivity %	Resistance %
Clindomycin	50	50
Amikacin	70	30
Amoxycillin	60	40
Cephalexin	40	60
Tetracycline	65	35
Erythromycin	48	52
Cephalothin	50	50
Gentamicin	50	50
Kanamycin	38	62
Cloxacillin	0	100
Rifampin	83	17
Neomycin	20	80
Benzoyl-Peroxide	75	25
Clindamycin + Benzoyl-Peroxide	63	27
Erythromycin + Benzoyl-Peroxide	67	23

## Discussion

In this study, more *Staphylococcus aureus* and *Micrococcus spp* were found in aerobic cultures while more *Staphylococcus aureus* and *Propionibacterium acne* responsible for acne, were found in anaerobic cultures. Since the most frequent bacteria isolated from acne patients were *Staphylococcus aureus*, it is possible that acne acne vulgaris is mainly caused by *Staphylococcus aureus* rather than *Propionibacterium cane*.^13^,^14^ This is in contrast to some reports which implicated both *Staphylococcus epidermidis* and *Propionibacterium acnes* as bacteria causing acne vulgaris.^15^–^17^ It may be concluded that geographical regions affect the bacteria involved in acne vulgaris.^10^ Since bacterial resistance to conventional antibiotics such as erythromycin and tetracycline were reported to have an increasingly trend,^10^ research on finding the effective antibiotics seems indispensable. *In vitro* inhibition of *Propionibacterium acne* by a bacteriocin-like inhibitory substance (BLIS-Like substance) produced by *Streptococcus salivarius* was previously reported and in some studies BLIS was suggested for its anti-*Propionibacterium acne a* ctivity in the treatment of acne patient.^18^ In this geographical area with *Staphylococcus aureus* as primary casual agent in acne development, *Staphylococcus aureus* was resistant to tetracycline, erythromycin and clindamycin which is consistent to reports by some other investigators,^19^–^21^ but was highly sensitive to Rifampin. On the basis of these results, we suggest that rifampin is a suitable antibiotic for acne patients, but to achieve a better result, combination of rifampin with other antibiotics seems necessary. Also we suggest an *in vivo* study to be performed for better evaluation acne vulgaris treated by rifampin.

## References

[1] Hanna S, Sharma J, Klotz J (2003). Acne vulgaris: More than skin deep. Dermatol Online J.

[2] Webster GF, Leyden JJ, Nilsson UR (1979). Complement activation in acne vulgaris: Consumption of complement by comedones. Infect Immun.

[3] Simpson NB, Burn T, Breathnach S, Cox N, Grifiths C (2004). Disorders of the sebaceous glands. Rook's Textbook of Dermatology.

[4] Firooz A, Sarhangnejad R, Davoudi SM, Nassiri-Kashani M (2005). Acne and smoking: Is there a relationship?. BMC Dermatol.

[5] Adebamowo CA, Spiegelman D, Berkey CS, Danby FW, Rockett HH, Colditz GA (2006). Milk consumption and acne in adolescent girls. Dermatol Online J.

[6] Smith RN, Mann NJ, Braue A, Mäkeläinen H, Varigos GA (2007). A low-glycemic-load diet improves symptoms in acne vulgaris patients: A randomized controlled trial. Am J Clin Nutr.

[7] Gollnick HP, Krautheim A (2003). Topical treatment in acne: current status and future aspects. Dermatology.

[8] Stein RH, Lebwohl M (2001). Acne therapy: Clinical pearls. Semin Cutan Med Surg.

[9] Zaenglein AL, Thiboutot DM, Bolognia JL, Jorizzo JL, Rapini RP (2003). Acne vulgaris. Dermatology.

[10] Ashkenazi H, Malik Z, Harth Y, Nitzan Y (2003). Eradication of Propionibacterium acnes by its endogenic porphyrins after illumination with high intensity blue light. FEMS Immunol Med Microbiol.

[11] Ross JI, Snelling AM, Eady EA, Cove JH, Cunliffe WJ, Leyden JJ (2001). Phenotypic and genotypic characterization of antibiotic-resistant Propionibacterium acnes isolated from acne patients attending dermatology clinics in Europe, the USA, Japan and Australia. Br J Dermatol.

[12] Baron EJ, Finegold SM (1990). Methods for testing antimicrobial effectiveness. Diagnosis microbiology.

[13] Toyoda M, Morohashi M (1998). An overview of topical antibioticforacne treatment. Dermatology.

[14] Rodriguez-Cavallini E, Vargas-Dengo P (2004). Etiologyia bacteriana y susceptiblidad a antibioticos en pacientes con acne. Rev Biomed.

[15] Thiboutot D (2000). New treatments and therapeutic strategies for acne. Arch Fam Med.

[16] Leyden JJ (2002). Effect of topical benzoyl peroxide-clindamycin versus topical cindamycin and *Propionibacterium acnes*. Cutis.

[17] Ross JI, Eady EA, Cove JH, Ratyal AH, Cunliffe WJ (1998). Resistance to erythromycin and clindamycin in cutaneous propionibacteria is associated with mutations in 23S rRNA. Dermatology.

[18] Bowe WP, Filip JC, DiRienzo JM, Volgina A, Margolis DJ (2006). Inhibition of propionibacterium acnes by bacteriocin-like inhibitory substances (BLIS) produced by Streptococcus salivarius. J Drugs Dermatol.

[19] Cunliffe WJ, Baron SE, Coulson IH (2001). A clinical and therapeutic study of 29 patients with infantile acne. Br J Dermatol.

[20] Noyon V, Legallou F, Richet H, Dreno B (1998). The resistance of Propionibacterium acnes and Staphylococcus epidermidis to cyclones. Ann Dermatol Venereol.

[21] Tan HH, Goh CL, Yeo MG, Tan ML (2001). Antibiotic sensitivity of Propionibacterium acnes isolates from patients with acne vulgaris in a tertiary dermatological referral centre in Singapore. Ann Acad Med Singapore.

